# ITO Thin Films for Low-Resistance Gas Sensors

**DOI:** 10.3390/ma16010342

**Published:** 2022-12-29

**Authors:** Aleksei V. Almaev, Viktor V. Kopyev, Vadim A. Novikov, Andrei V. Chikiryaka, Nikita N. Yakovlev, Abay B. Usseinov, Zhakyp T. Karipbayev, Abdirash T. Akilbekov, Zhanymgul K. Koishybayeva, Anatoli I. Popov

**Affiliations:** 1 Research and Development Center for Advanced Technologies in Microelectronics, National Research Tomsk State University, 634050 Tomsk, Russia; 2 Fokon LLC, 248035 Kaluga, Russia; 3 Ioffe Institute of the Russian Academy of Sciences, 194021 Saint Petersburg, Russia; 4 Faculty of Physics and Technical Sciences, L.N. Gumilyov Eurasian National University, Astana 010008, Kazakhstan; 5 Institute of Solid State Physics, University of Latvia, 8 Kengaraga Str., LV-1063 Riga, Latvia

**Keywords:** indium tin oxide, thin films, gas sensors

## Abstract

Indium tin oxide thin films were deposited by magnetron sputtering on ceramic aluminum nitride substrates and were annealed at temperatures of 500 °C and 600 °C. The structural, optical, electrically conductive and gas-sensitive properties of indium tin oxide thin films were studied. The possibility of developing sensors with low nominal resistance and relatively high sensitivity to gases was shown. The resistance of indium tin oxide thin films annealed at 500 °C in pure dry air did not exceed 350 Ohms and dropped by about 2 times when increasing the annealing temperature to 100 °C. Indium tin oxide thin films annealed at 500 °C were characterized by high sensitivity to gases. The maximum responses to 2000 ppm hydrogen, 1000 ppm ammonia and 100 ppm nitrogen dioxide for these films were 2.21 arbitrary units, 2.39 arbitrary units and 2.14 arbitrary units at operating temperatures of 400 °C, 350 °C and 350 °C, respectively. These films were characterized by short response and recovery times. The drift of indium tin oxide thin-film gas-sensitive characteristics during cyclic exposure to reducing gases did not exceed 1%. A qualitative model of the sensory effect is proposed.

## 1. Introduction

The expansion of hydrogen energy and the deterioration of air quality near urban infrastructure and industrial areas highlight the necessity to develop new gas sensors. Resistive sensors based on metal oxide semiconductors are advisable to use for gas detection in the air due to their high sensitivity, diminutiveness, low-cost fabrication and energy efficiency [1,2,3,4,5,6,7,8]. However, the metal oxide semiconductor’s sensitive layer resistance reaches tens or hundreds of MOhm and GOhm. This limits the use of standard power components and processing elements and significantly increases the costs of devices based on metal oxide semiconductor sensors and their energy consumption [9,10,11,12,13]. The nominal resistance of Figaro commercial tin dioxide (SnO_2_) sensitive elements lies in the range from 1 kOhm to 10 kOhm when exposed to 100 ppm H_2_ or from 1 kOhm to 200 kOhm in pure air. A low nominal resistance not higher than or within the specified ranges for commercial sensors is necessary to achieve for developing a competitive device. Commercial SnO_2_-sensitive elements are obtained by thick-film and ceramic technologies [14]. At the same time, thin-film sensing elements are of significant interest, primarily due to the high ratio between the surface area and the bulk of the semiconductor. This ratio allows the enhancement of the effect of gas molecule chemisorption on the electrically conductive properties of the material. The thin-film technology allows low-cost fabrication and the possibility of combining it with standard microelectronic technologies [15,16,17,18,19].

It is difficult to combine low nominal resistance and high sensitivity to gases for thin films of metal oxide semiconductors. In general, the sensory effect of metal oxide semiconductors consists of the interaction of gas molecules with previously chemisorbed oxygen on the semiconductor surface. During their chemisorption on the metal oxide semiconductor surface, oxygen molecules capture electrons from its conduction band. This process leads to an increase in the resistance of the *n*-type semiconductor. During chemisorption, molecules of reducing gases interact with previously chemisorbed oxygen. The captured electrons return to the semiconductor during this process, and its resistance decreases. The sensor response *S* is *~* exp(*N*_i_^2^/*N_d_*) [20,21,22] at *D_g_* >> *L_D_*, where *N_i_* is the surface density of chemisorbed oxygen; *N_d_* is the concentration of donor impurities; *D_g_* is the semiconductor grain diameter; and *L*_D_ is the Debye length. The *N*_i_^2^/*N_d_* ratio can be varied by adding bulk and surface impurities. In this case, the drift of sensor characteristics increases at high operating temperatures [23]. The necessary *N*_i_^2^/*N_d_* ratio providing high gas sensitivity and electrical conductivity can be achieved by using a mixture of metal oxide semiconductors [24]. The first metal oxide semiconductor provides high electrical conductivity due to its fundamental properties, and the second provides high sensitivity to gases due to its catalytic activity. Such a material is a mixture of indium oxide (In_2_O_3_) and SnO_2_, with 5–15% of SnO_2_ corresponding to indium tin oxide (ITO) [25,26,27,28,29,30,31].

Pen plotter printing [25], impregnation [26], magnetron sputtering (MS) [27,28,29,30] and plasma-chemical [31] methods have been used to obtain ITO thin films in order to study their gas-sensitive properties. The thicknesses of ITO thin films ranged from 20 nm to 600 nm. The sensitivity of ITO thin films to gases largely depended on the method of their deposition. So, ITO films with 5–10 at.% Sn obtained by pen plotter printing showed the highest responses to carbon monoxide (CO) at the operating temperature *T* = 200 °C by impregnation and to hydrogen (H_2_) at *T* = 320 °C, and MS-deposited films were characterized by high responses to ammonia (NH_3_) at *T* = 150 °C and nitrogen dioxide (NO_2_) at *T* = 300 °C. The MS method allows variations in many parameters during the deposition of films that affect their electrically conductive and gas-sensitive properties. An optimal ratio between the nominal resistance of the film and its sensitivity to gases can be relatively easily achieved by means of MS [27,28,29,30]. In Refs. [27,29], the responses to 100 ppm NH_3_ and 1000 ppm H_2_ at *T* = 150 °C were 24.1 and 11, respectively, and the resistance in pure air did not exceed 35.6 kOhm. In Ref. [31], the resistance in pure air in the range of *T* = 100–500 °C varied in the range of 10^3^–10^4^ Ohm, and the maximum responses to H_2_ and NO_2_ were 8 and ~160 at *T* = 400 °C and 300 °C, respectively.

Thus, MS-deposited ITO thin films are of interest for the development of highly sensitive gas sensors with low nominal resistance. It is worth noting the lack of studies on structures based on ITO with a resistance below 10^3^ Ohms or on the order of several hundred Ohms. This research is devoted to the study of the structural, electrically conductive and gas-sensitive properties of MS-deposited ITO thin films with extremely low resistance.

## 2. Materials and Methods

ITO thin films were obtained by the direct-current MS of the oxide target in oxygen–argon plasma using Edwards A-500 (Edwards, Sanborn, NY, USA) equipment. Polished aluminum nitride (AlN) ceramic wafers with a thickness of 150 µm were used as substrates. The wafers were treated in sulfuric acid, isopropyl alcohol and deionized water before the deposition of ITO films. The substrate temperature corresponded to room temperature in the process of ITO film deposition. The working pressure and power were 7 × 10^−3^ mbar and 70 W, respectively. The oxygen concentration in the oxygen + argon mixture was 10 ± 0.5 vol.%. The distance between the substrate and the target was 70 mm. The thickness of the ITO films was 180 nm for a deposition time of 20 min. The as-prepared films were annealed for 60 min in the air at temperatures *T_ann_* = 500 °C and 600 °C. We denote the series of ITO thin films annealed at *T_ann_* = 500 °C as ITO-500 and those annealed at *T_ann_* = 600 °C as ITO-600.

**Figure 1:**
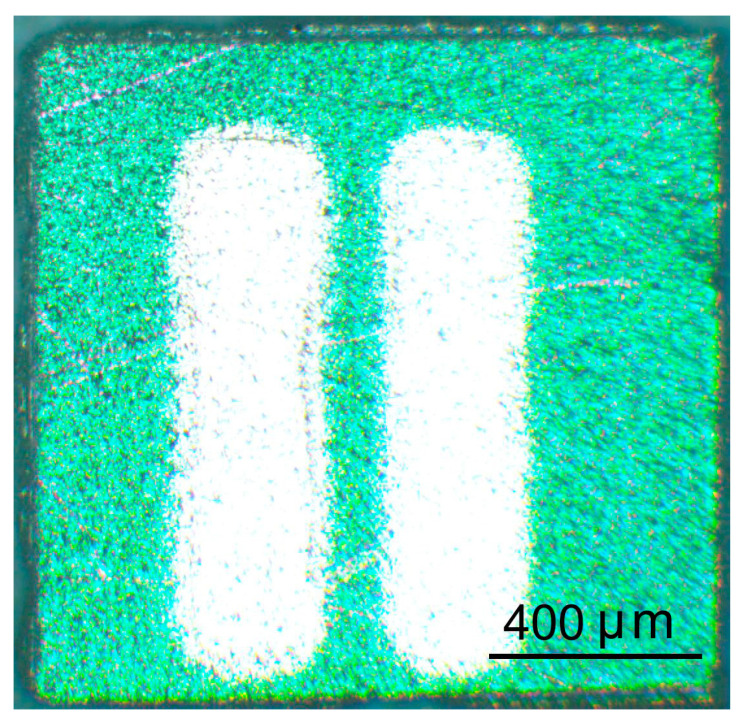
Microscopic photo of the sensor element based on ITO thin film.

Pt plane-parallel contacts were deposited on the ITO thin film surface through a mask to measure the electrically conductive and gas-sensitive properties. The interelectrode distance was 150 μm. A photo of the sensor element produced by means of a metallographic microscope Altami MET 6 C (Altami LLC, Saint Petersburg, Russia) is shown in Figure 1. Before measuring the electrically conductive and gas-sensitive properties, the samples were preliminarily heated at *T* = *T_ann_* − 50 °C in a stream of pure dry air to stabilize the properties of the contacts, to regenerate the surface and to activate oxygen chemisorption.

The surface morphology of the ITO thin films was studied using an atomic force microscope (AFM) (Solver HV from NT-MDT). Energy-dispersive X-ray (EDX) spectroscopy of ITO films was carried out using a Phenom ProX scanning electron microscope (Thermo Fisher Scientific，Shanghai, China) with special detectors at an accelerating voltage of 15 kV. This EDX mode with an accelerating voltage of 15 kV is adequate for the study of ITO films with a thickness of 180 nm [32,33,34,35]. X-ray diffraction analysis (XRD) was performed to determine the phase composition of the thin films. XRD measurements were carried out using a diffractometer (XRD 6000, Shimadzu, Tokyo, Japan) with CuKα radiation. The X-ray source wavelength was 1.54 Å. Transmission spectra were measured for ITO thin films deposited on a polished c-plane sapphire substrate with a thickness of 150 µm. A DH-2000 irradiation source based on deuterium and tungsten halogen lamps and Ocean Optics spectrometric systems were used to measure the transmission spectra of films at room temperature.

Measurements of the current–voltage (*I*–*V*) characteristics and the time dependence of the sample resistance under exposure to various gases were carried out using a Keithley 2636A source-meter and a hermetic Nextron MPS-CHH (Nextron) microprobe station. This microprobe station allows the measurement of the electrically conductive characteristics of films in the temperature range from room temperature to 750 °C with an accuracy of *T* ± 0.1 °C. Measurements were carried out in dark conditions in a stream of pure dry air or in a gas mixture of pure dry air + target gas. H_2_, NH_3_, CO, NO_2_ and methane (CH_4_) were selected as target gases. The flow rate of gas mixtures through the measurement chamber (100 cm^3^ in volume) of the microprobe station was maintained at 1000 cm^3^/min. The source of pure dry air was a special generator. The concentration of the target gas in the mixture was controlled by a gas mixture generator with Bronkhorst mass flow regulators. The relative error of the gas flow rate did not exceed 1.5%. The voltage applied to the sample electrodes was 2 V.

## 3. Results

### 3.1. Structural Properties of ITO Thin Films

**Figure 2:**
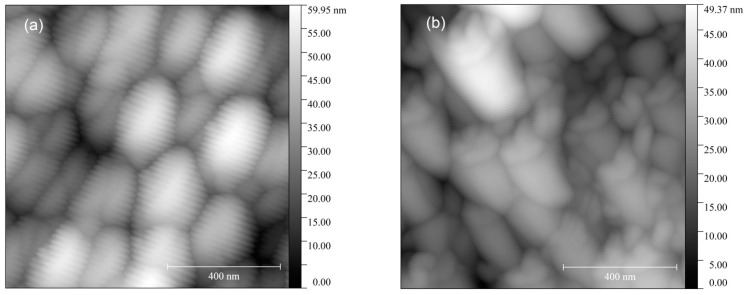
AFM images of the ITO thin-film surfaces annealed at *T_ann_* = 500 °C (**a**) and 600 °C (**b**).

The microrelief of the ITO-500 film surface is represented by small grains with sizes of 40–100 nm, which form large agglomerates with sizes up to 350 nm (Figure 2). An increase in *T_ann_* to 600 °C leads to an increase in the size of small grains to 80–140 nm. At the same time, the sizes of agglomerates vary slightly.

**Figure 3:**
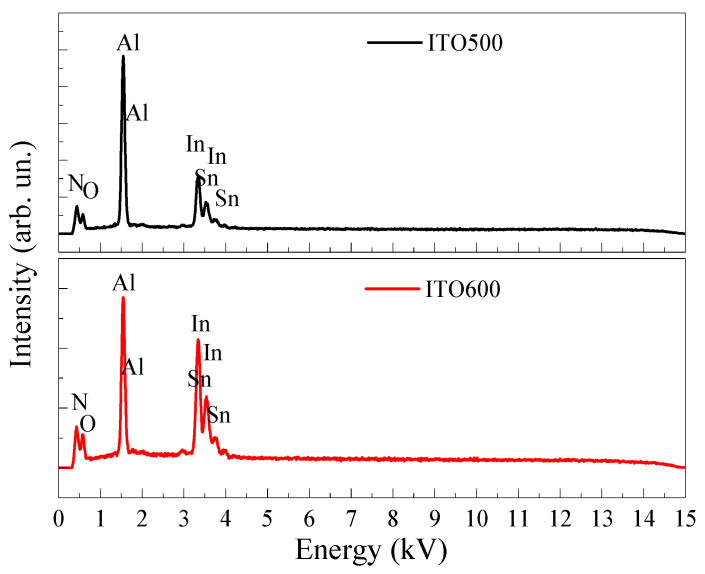
EDX spectra of ITO thin films annealed at *T_ann_* = 500 °C and 600 °C.

The EDX spectra (Figure 3) show intense peaks of Al and N from the substrate and In, Sn and O from the film deposited on top of the substrate. An increase in *T_ann_* leads to an increase in the contents of In and Sn in the films due to a decrease in the content of O (Table 1). In Ref. [36], it was shown that in as-deposited ITO thin films, Sn forms complexes with O on the grain surface and intergrain space due to the excess of the latter. Annealing leads to the destruction of these complexes and enhances the diffusion of Sn into In_2_O_3_. The increase in grain sizes with *T_ann_* observed by AFM can lead to a decrease in the density of Sn–O complexes on the film surface.

**Figure 4:**
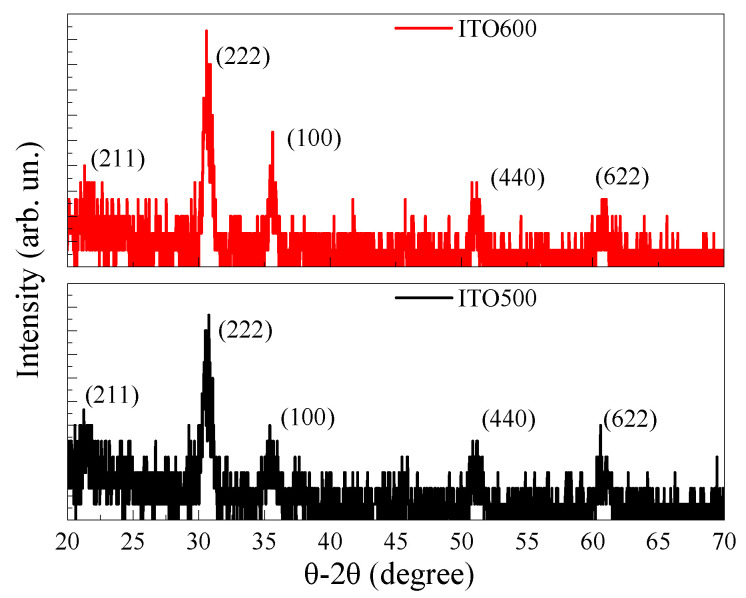
XRD spectra of ITO thin films annealed at *T_ann_* = 500 °C and 600 °C.

The XRD spectra of the annealed ITO thin films are characterized by the presence of low-intensity peaks that can be associated with the (211), (222), (100), (440) and (622) crystallographic planes of the cubic In_2_O_3_ phase (Figure 4). The spectra were measured at a shallow angle. For this reason, it was not possible to detect peaks from the substrate. The content of SnO_2_ is not enough to detect its phase in ITO thin films using XRD. Similar XRD spectra were observed earlier for ITO thin films [25,29,30,31]. An increase in *T_ann_* leads to an increase in the intensity of the peaks without changing their position. The estimation of the crystallite sizes *D* using the Scherrer equation was made for the most intense peaks associated with the (222) and (100) crystallographic planes. For ITO-500 films, *D* = 22.6 nm and 20.9 nm, respectively, for these peaks. An increase in *T_ann_* to 600 °C leads to an increase in the *D* to 26.1 nm and 28.0 nm.

**Figure 5:**
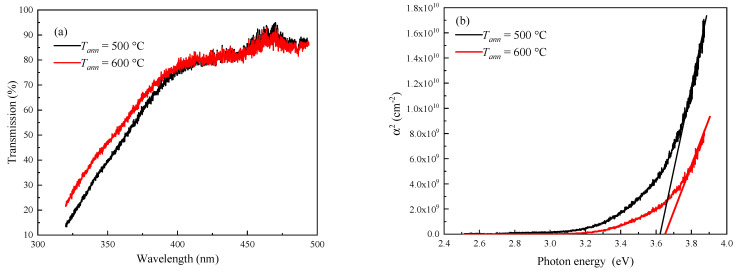
Optical transmission spectra (**a**) and *α*^2^ versus the photon energy (**b**) for ITO thin films annealed at *T_ann_* = 500 °C and 600 °C.

The optical transmission spectra of ITO films annealed at *T_ann_* = 500 °C and 600 °C are shown in Figure 5a. The transmission at wavelengths *λ* > 400 nm exceeds 80%, which is characteristic of ITO films [37]. In this wavelength region, the annealing temperature does not significantly affect the transmission values. The observed peak in the range of *λ* = 450–475 nm is caused by interference phenomena in the film–substrate system. With a decrease in *λ* from 400 nm to 320 nm, the transmission decreases due to the band-to-band absorption. In this *λ* range, the transmittance of ITO films annealed at *T_ann_* = 600 °C is slightly higher. Optical absorption spectra can be analyzed fairly accurately from the curves of *α*^2^ vs. photon energy *hν* (Figure 5b) characterized for the direct-band-gap semiconductor, where *α* is the absorption coefficient. The band gaps *E_g_* were 3.62 eV and 3.65 eV for the ITO-500 and ITO-600 films, respectively. The obtained values of *E_g_* and those indicated in Refs. [37,38] for MS-deposited ITO thin films are practically the same.

Thus, the studied films are a mixture of indium and tin oxides, with the content of the latter at a level corresponding to ITO. It will be shown below that ITO thin films are of interest for the development of low-resistance gas sensors. For this reason, in this work, we have focused on the gas-sensitive properties of the material. In the future, we plan to conduct detailed studies of the structural properties of ITO thin films by means of X-ray photoelectron and Raman spectroscopies, just as was implemented in Refs. [39,40].

### 3.2. The Electrically Conductive Properties of ITO Thin Films in Pure Dry Air

**Figure 6:**
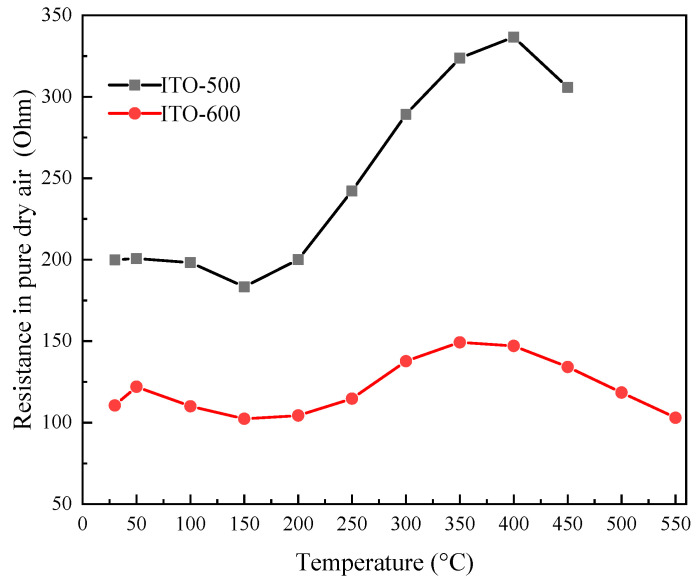
Temperature dependence of the ITO thin-film resistance in pure dry air at *T_ann_* = 500 °C and 600 °C.

Figure 6 shows the temperature dependence of the ITO thin-film resistance in pure dry air, *R_air_*. *R_air_* decreases slightly with an increase in *T* in the range from 50 °C to 150 °C regardless of *T_ann_*, which is characteristic of a semiconductor. *R_air_* increases with a further increase in *T* to 350–400 °C. The active chemisorption of oxygen on the thin-film surface and the transition of chemisorbed oxygen from the molecular form O_2_^−^ to atomic O^−^ take place in this *T* range [20,21,22]. The atomic form of chemisorbed oxygen is the most active in interacting with reducing gases. An increase in resistance with *T* is characteristic of thin films. The surface determines the electrically conductive properties to a greater extent than the bulk of thin films. During chemisorption, oxygen captures electrons from the conduction band of a semiconductor [20,21,22]. Increasing *T* from 200 °C to 350–400 °C leads to a rise in *N*_i_ and a drop in the electron concentration *n* in the film. At *T* > 350–400 °C, the desorption of O^−^ manifests, and *R_air_* drops again with temperature. The observed increase in *R_air_* correlates with the results of Hall measurements for polycrystalline ITO thin films [41]. Electron mobility decreases slightly with *T* due to ionized impurity scattering and grain boundary scattering. The electron concentration in the *T* range from 25 °C to 200 °C does not significantly change and significantly decreases with an increase in *T* to 500 °C. The decrease in *n* is due to the interaction of the ITO film with oxygen.

An increase in *T_ann_* of ITO thin films leads to a decrease in *R_air_*, mainly due to the observed increase in grain size [42]. It was shown in Refs. [36,43] that an increase in the grain size of ITO thin films with *T_ann_* leads to a decrease in film resistance due to an increase in carrier mobility. Increasing the grain size of the studied ITO thin films was confirmed by our results of ASM and XRD. The ITO thin-film resistance at different temperatures does not exceed 350–400 Ohms. Thus, sensors based on the studied films have low nominal resistance.

### 3.3. Gas-Sensitive Properties of ITO Thin Films

**Figure 7:**
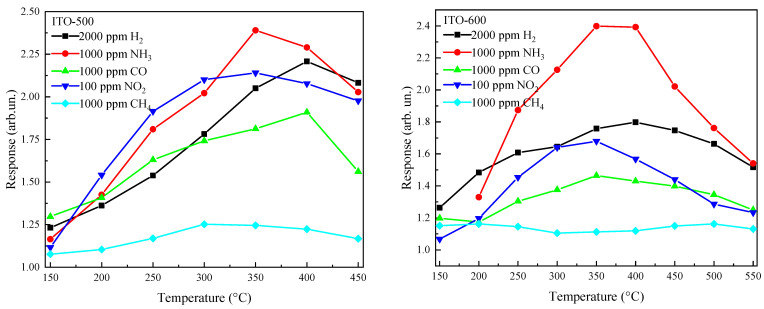
Temperature dependence of ITO thin-film responses to fixed concentrations of various gases at *T_ann_* = 500 °C and 600 °C.

Figure 7 shows the dependence of the responses to fixed concentrations of various gases on the temperature for ITO thin films annealed at *T_ann_* = 500 °C and 600 °C. Exposure to the reducing gases H_2_, NH_3_, CO and CH_4_ in the temperature ranges of 150–450 °C and 150–550 °C for ITO-500 and ITO-600 thin films, respectively, leads to a reversible decrease in their resistance. In the region of low temperatures, *T* ≤ 100 °C, the resistance of the samples after their exposure to the gases is practically unrecovered. The following relation was selected for the response to reducing gases:

*S*_1_ = *R*_air_/*R*_g_, (1)

where *R_g_* is the ITO thin-film resistance in a gas mixture of pure dry air + target gas (H_2_, NH_3_, CO and CH_4_). The exposure to NO_2_ leads to a reversible increase in ITO thin-film resistance. The following relation was used for the response to NO_2_:

*S*_2_ = *R*_NO2_/*R*_air_, (2)

where *R*_NO2_ is the ITO thin-film resistance in a gas mixture of pure dry air + NO_2_. The curves in Figure 7 are characterized by the presence of maxima *S_MAX_* at a certain temperature, *T_MAX_.* It is advisable to choose *T_MAX_* as the operating temperature. *T_MAX_* and *S_MAX_* for various gases for ITO thin films annealed at *T_ann_* = 500 °C and 600 °C are presented in Table 2, where *n_g_* is the target gas concentration. The highest responses to NH_3_ and NO_2_ are observed for ITO-500 thin films. An increase in *T_ann_* leads to a decrease in the responses of ITO thin films to all gases except NH_3_. ITO thin films exhibit relatively low responses to CH_4_. Table 2 also shows the resistance in pure air at *T* = *T_MAX_*. The observed variations in *R*_air_ are due to variations in the tin concentration in the ITO thin films (Table 1).

**Figure 8:**
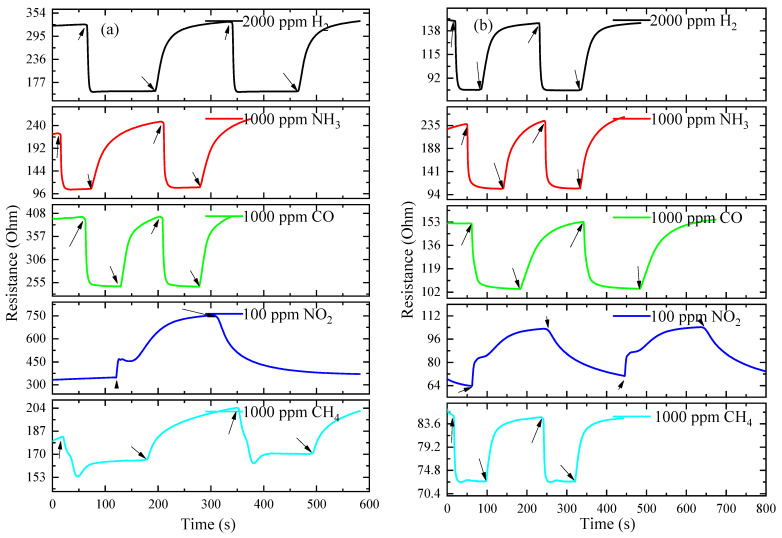
Time dependence of ITO thin-film resistance at *T_ann_* = 500 °C (**a**) and 600 °C (**b**) when exposed to various gases and *T = T_MAX_*.

The time dependence of the ITO thin-film resistance at *T_ann_* = 500 °C and 600 °C under exposure to various gases at *T = T_MAX_* is shown in Figure 8. The falling and rising regions of the ITO thin-film resistance under exposure to reducing gases are approximated by the following functions, respectively:

*R*(*t*) = *R*_g_ + *A*_1_exp[−*t*/τ_1_], (3)

*R*(*t*) = *R*_air_ − *B*_2_exp[−*t*/τ_2_], (4)

where *A*_1_ and *B*_2_ are constants; *t* is time; and *τ*_1_ and *τ*_2_ are time constants for the falling and rising regions of the ITO thin-film resistance under exposure to reducing gases. The rising region of ITO thin-film resistance under exposure to NO_2_ is approximated by the function:

*R*(*t*) = *R*_NO2_ − *A*_NO2_exp[−*t*/τ_3_], (5)

and the falling-region resistance after exposure to NO_2_ is approximated by the function:

*R*(*t*) = *R*_air_ + *B*_NO2_exp[−*t*/τ_4_], (6)

where *A*_NO2_ and *B*_NO2_ are constants; *τ*_3_ and *τ*_4_ are time constants for the rising and falling regions of the ITO thin-film resistance under exposure to NO_2_.

**Figure 9:**
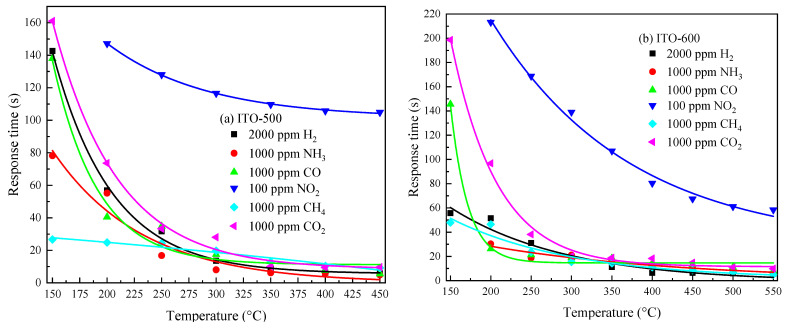
Temperature dependence of response times for ITO thin films annealed at *T_ann_* = 500 °C (**a**) and 600 °C (**b**) under exposure to various gases.

**Figure 10:**
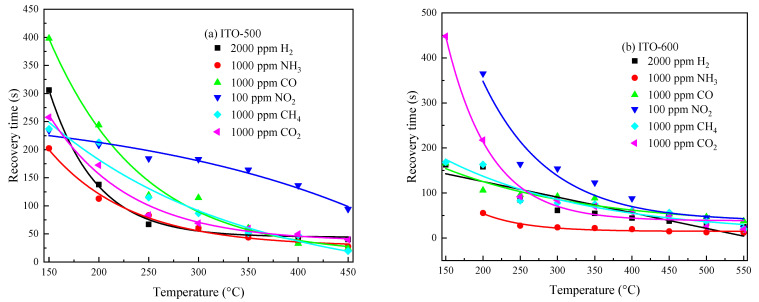
Temperature dependence of recovery times for ITO thin films annealed at *T_ann_* = 500 °C (**a**) and 600 °C (**b**) under exposure to various gases.

*τ*_1_, *τ*_3_ and *τ*_2_, *τ*_4_ are defined by the relaxation times *τ* of the adsorption/desorption processes of gas molecules on the semiconductor surface: *τ* ~ exp[(*E_D_–E_A_*)/(2*kT*)] [44], where *E_A_* and *E_D_* are the activation energies of the adsorption and desorption of gas molecules on the semiconductor surface, respectively; *k* is the Boltzmann constant. *τ* and, consequently, *τ*_1_, *τ*_3_ and *τ*_2_, *τ*_4_ decrease sharply with *T*. It can be seen from Expressions (3) and (4) that at *t* ≥ 2.3*τ*_1_ and *t* ≥ 2.3*τ*_2_, stationary values of *R_g_* and *R_air_* are achieved. The response time *t*_res_ = 2.3*τ*_1_ and the recovery time *t*_rec_ = 2.3*τ*_2_ can be used to evaluate the operation speed of ITO thin films under exposure to reducing gases. *t_res_* = 2.3*τ*_3_ and *t_rec_* = 2.3*τ*_4_ under exposure to NO_2_. The temperature dependence of the response and recovery times for ITO thin films is shown in Figure 9 and Figure 10, respectively.

The response and recovery times drop exponentially with *T*. The response times of ITO thin films for all reducing gases at *T* ≥ 350 °C do not exceed 20 s, and the recovery times do not exceed 100 s. The longest *t_res_* and *t_rec_* are observed under exposure to NO_2_, which is caused by the large value of the binding energy of this molecule to the surface [45].

**Figure 11:**
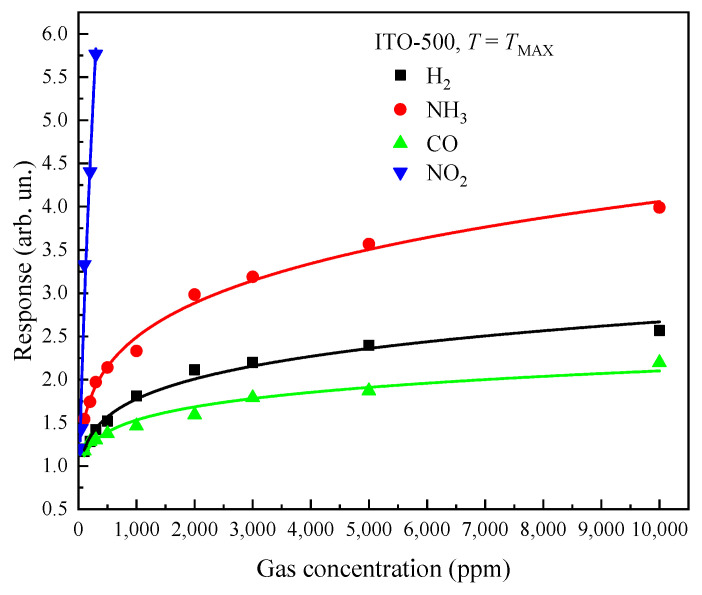
Dependence of ITO-500 thin-film responses on concentrations of H_2_, NH_3_, CO and NO_2_ at *T* = *T_MAX_*.

ITO thin films annealed at *T_ann_* = 500 °C are characterized by the highest responses to H_2_, NH_3_, CO and NO_2_. Therefore, only these films will be further considered. Figure 11 shows the dependence of the responses on H_2_, NH_3_, CO and NO_2_ concentrations for ITO-500 thin films at *T = T_MAX_*. The response increases with the concentration according to the power law, *S*_1,2_ ~ *n*_g_*^m^*, where *m* is the power index. The *m* values for H_2_, NH_3_, CO and NO_2_ at *T = T_MAX_* are compared in Table 3.

**Figure 12:**
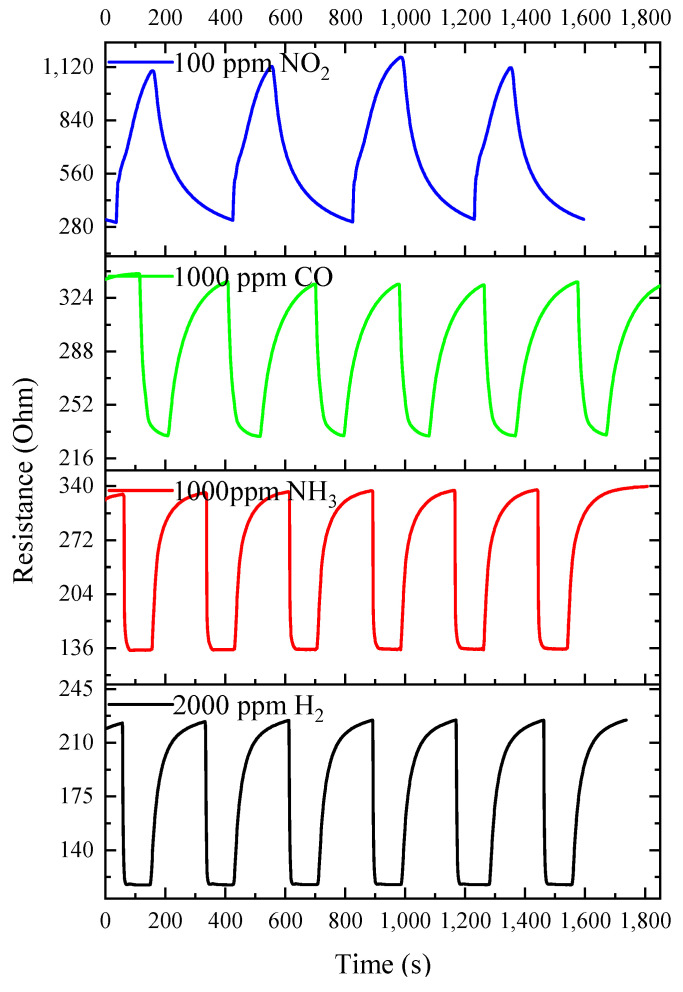
Time dependence of the ITO-500 thin-film resistance under cyclic exposure to H_2_, NH_3_, CO and NO_2_ at *T* = *T_MAX_*.

**Figure 13:**
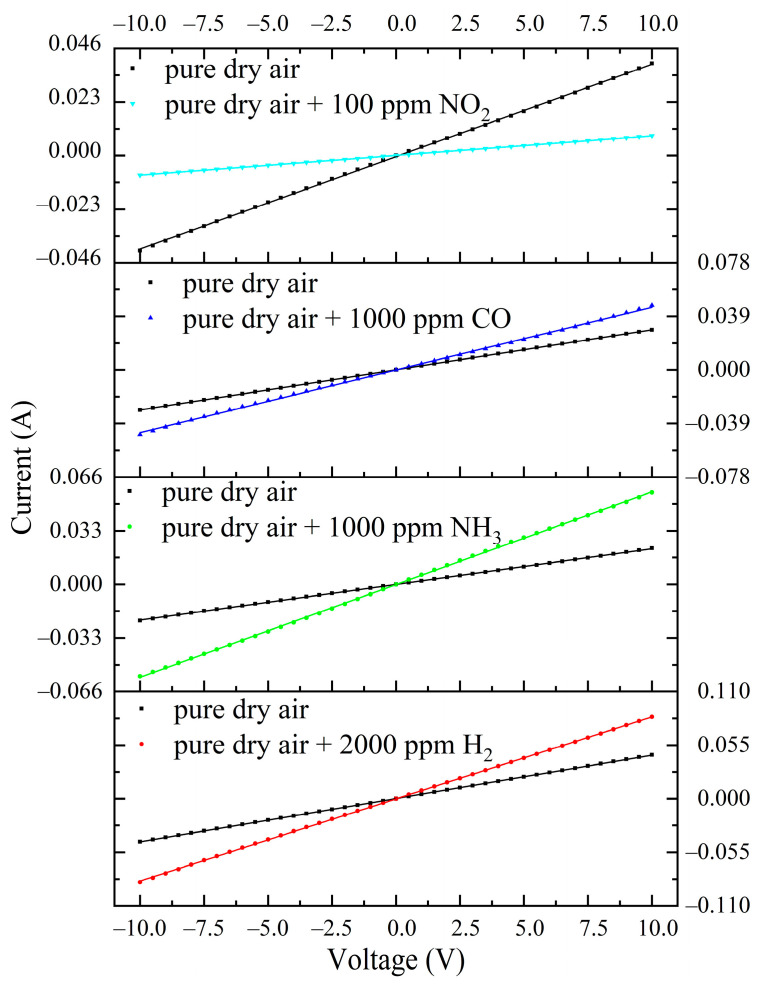
*I*–*V* characteristics of ITO-500 thin films in pure dry air and when exposed to H_2_, NH_3_, CO and NO_2_ at *T* = *T_MAX_*.

Figure 12 shows the time dependence of the resistance of ITO-500 thin films at *T = T_MAX_* and under cyclic exposure to H_2_, NH_3_, CO and NO_2_. Estimates have shown that the drift of ITO-500 thin-film characteristics is practically absent under exposure to reducing gases. The deviations of *R*_air_, *R*_g_ and *S*_1_ from the average values do not exceed 1%. Significant deviations of 5% occurred under exposure to NO_2_. The *I*–*V* characteristics of ITO-500 films in pure dry air and when exposed to H_2_, NH_3_, CO and NO_2_ are linear in the voltage range of −10–10 V (Figure 13). Exposure to gases leads to a change in the slope of the *I*–*V* characteristics due to a change in the ITO thin-film resistance.

Estimates have shown that *L*_D_ for ITO-500 thin films increases from 0.57 nm to 1.1 nm with an increase in *T* from 30 °C to 450 °C. Thus, the ratio *D_g_* >> *L_D_* was obtained, and an over-barrier conduction mechanism is manifested for ITO-500 thin films. In this case, the conductivity *G* of the thin film is as follows [20,21,22]:

*G*(*T*) = *G*_00_(*T*)exp[*e*φ*_s_*(*T*)/(*kT*)], (7)

where *G*_00_ is a parameter that weakly depends on changes in the atmospheric composition and is determined by the geometric dimensions of the ITO film and its electrophysical characteristics; *e*φ_s_ is the band-bending energy at the grain boundaries; φ_s_ is the surface potential; and *e* is the electron charge. In an atmosphere of air, oxygen molecules are chemisorbed on the surface of the semiconductor.

For an n-type semiconductor, oxygen chemisorption leads to the upward bending of energy bands [20,21,22]. The predominant form of chemisorbed oxygen in the range of *T* = 150–500 °C is O^−^ [46]. For metal oxide semiconductors, *e*φ*_s_* ~ *N*_i_^2^ and *e*φ*_s_* ~ *n*_O2_*^l^*, where *n*_O2_ is the oxygen concentration in the gas mixture; *l* is an index that depends on the adsorption properties of the semiconductor surface, adsorption centers, etc., with *l* < 1. Under exposure to reducing gases, the interaction between their molecules and previously chemisorbed O^-^ ions takes place, leading to decreases in *N*_i_ and *e*φ_s_. NO_2_ molecules are able to chemisorb onto free adsorption centers and, like oxygen, capture electrons from the conduction band of the semiconductor. This process leads to an additional increase in *e*φ_s_ ~ (*N*_i_ + *N*_NO2_)^2^, where *N*_NO2_ is the surface density of chemisorbed NO_2_. Changes in *G* when exposed to gases are caused mainly by changes in *e*φ_s_. Interactions between target gas molecules and O^−^ on the ITO surface can be described by the following reactions [47,48,49]:

H_2_ + O^−^ → H_2_O + e, (8)

2NH_3_ + 3O^−^ → N_2_ + 3H_2_O + 3e, (9)

CH_4_ + 4O^−^ → CO_2_ + 2H_2_O + 4e, (10)

CO + O^−^ → CO_2_ + e. (11)

NO_2_ + e^−^ → NO_2_^−^, (12)

As a result of Reactions (8)–(11), the conductivity of the semiconductor increases, and reaction products in the form of H_2_O, N_2_ and CO_2_ molecules are desorbed. Reactions (8)–(12) are the simplest possible and only fundamentally explain the observed sensory effect. Many reasonable variants of other reactions on the surface of metal oxide semiconductors have been proposed. Their consideration is not advisable to describe a qualitative model of the sensory effect for the studied ITO films.

In Ref. [26], the sensory mechanism of the In_2_O_3_ and SnO_2_ mixed-oxide system was studied in more detail. The tin oxide content in the mixture varied widely. It was found that with an increase in the content of SnO_2_ from 0 to 10 wt.%, the conductivity of the mixture increases, and the response to H_2_ decreases. This composition corresponds to the studied ITO thin film. The limit of SnO_2_ solubility in In_2_O_3_ is 10–15 wt.%. The electrically conductive and gas-sensitive properties of the In_2_O_3_ and SnO_2_ mixed-oxide system are determined by In_2_O_3_ up to these values of SnO_2_ concentration. The introduction of Sn into the In_2_O_3_ matrix leads to a decrease in the surface density of chemisorbed oxygen ions and the responses of the films to H_2_. With a further increase in the concentration of SnO_2_, the conductivity decreases, and the response to H_2_ increases. Thus, in our case, Sn (SnO_2_) acts as an additive to In_2_O_3_ and reduces the resistance of the film. The sensing properties of the studied ITO thin films are determined by In_2_O_3_.

Table 4 shows a comparison of the gas-sensitive characteristics of ITO thin films with a SnO_2_ content of 5–10%. The resistance *R*_air_ and resistivity ρ_air_ of ITO thin films in pure air were measured at room temperature. It is worth noting that for most ITO thin films, high responses to gases corresponded to high nominal resistance. As a rule, high-sensitivity ITO films are characterized by a low thickness, less than 90 nm. High responses were shown by ITO films with an island microstructure [28]. The diameter of the ITO islands was several tens of nanometers. Depositing ultra-thin continuous and homogeneous semiconductor films by means of the MS method is a difficult task due to its peculiarities [50]. Nevertheless, reducing the film thickness is a promising method for enhancing gas sensitivity. The resistance and response to gases of the ITO films are significantly reduced at the high thickness of 300 nm [31]. The main advantage of the studied ITO thin films is the possibility of developing sensors with low nominal resistance (***R*_air_**) and relatively high sensitivity to gases (H_2_, NH_3_ and NO_2_).

## 4. Conclusions

The structural, optical, electrically conductive and gas-sensitive properties of magnetron-sputtered ITO thin films on AlN ceramic substrates annealed in air for 60 min at temperatures of 500 °C and 600 °C were investigated. The microrelief of ITO films annealed at 500 °C was represented by grains with dimensions of 40–100 nm, which form agglomerates up to 350 nm in size. The tin content in these films was 4.10–5.15 wt.%, and the band-gap energy was 3.62 eV. An increase in the annealing temperature to 600 °C led to increases in the size of grains to 80–140 nm, in the tin content to 4.97–6.15 wt.%, and in the band-gap energy to 3.65 eV. In pure dry air, the ITO thin-film resistance annealed at 500 °C does not exceed 350 Ohms, and the resistance of films annealed at 600 °C dropped by about 2 times. Sensitivity to H_2_, NH_3_, CO, NO_2_ and CH_4_ was studied in the operating temperature ranges of 150–450 °C and 150–550 °C for ITO films annealed at 500 °C and 600 °C, respectively. ITO films annealed at 500 °C were characterized by higher sensitivity to gases. The maximum responses to 2000 ppm H_2_, 1000 ppm NH_3_ and 100 ppm NO_2_ for these films were 2.21, 2.39 and 2.14 at operating temperatures of 400 °C, 350 °C and 350 °C, respectively. An increase in the annealing temperature led to a decrease in the gas sensitivity of the films. At operating temperatures of at least 350 °C, the films were characterized by short response and recovery times that did not exceed 20 s and 100 s, respectively, under exposure to reducing gases. The drift of the ITO film characteristics under cyclic exposure to reducing gases did not exceed 1%. A qualitative model of the sensory effect was proposed. The gas sensitivity of the ITO films was determined mainly by In_2_O_3_. SnO_2_ acted only as an additive, a source of Sn donor impurities. The possibility of developing sensors with low nominal resistance and relatively high sensitivity to gases was shown.

## Figures and Tables

**Table 1:** The content of elements in ITO thin films annealed at *T_ann_* = 500 and 600 °C.

*T_ann_* (°C)	Element Content (wt.%)		
	In	Sn	O
500	55.26–56.93	4.10–5.15	38.82–39.72
600	58.54–60.65	4.97–6.15	34.13–36.12

**Table 2:** Values of the maximum response to various gases and temperatures of the maximum response for ITO thin films annealed at *T_ann_* = 500 °C and 600 °C.

Gas	*n_g_* (ppm)	ITO-500			ITO-600		
		*T_MAX_* (°C)	*R_air_* (Ohm)	*S_MAX_* (arb. un.)	*T_MAX_* (°C)	*R_air_* (Ohm)	*S_MAX_* (arb. un.)
H_2_	2000	400	336.69	2.21	400	144.14	1.80
NH_3_	1000	350	259.87	2.39	350	210.01	2.40
CO	1000	400	370.14	1.91	350	154.17	1.46
NO_2_	100	350	337.55	2.14	350	62.40	1.68
CH_4_	1000	300	211.98	1.25	500	94.87	1.16

**Table 3:** Index *m* of ITO-500 thin films under exposure to various gases and at *T* = *T_MAX_*.

Gas	H_2_	NH_3_	CO	NO_2_
*m*	0.19 ± 0.01	0.21 ± 0.01	0.14 ± 0.01	0.72 ± 0.07

**Table 4:** Comparison of the gas-sensitive characteristics of ITO thin films.

*R_air_* (Ohm)	ρ*_air_* (Ohm × cm)	*T* (°C)	*n_g_* (ppm)	*S* (arb. un.)	Refs.
***H*_2_**					
-	7.5	250	100	1.1	[25]
~10^4^	-	150	1000	11	[29]
10^5^–10^6^	10^3^	100	100	6	[28]
-	2.47 × 10^−3^	350	400	1.55–1.7	[31]
10^3^	-	320	1100	25	[26]
200	3.6 × 10^−6^	400	2000	2.21	This work
***NH*_3_**					
-	~3	150	100	2	[25]
~10^4^		150	1000	24	[29]
10^5^–10^6^	10^3^	100	100	2	[28]
200	3.6 × 10^−6^	350	1000	2.39	This work
***NO*_2_**					
10^5^–10^6^	10^3^	100	200	80	[28]
10^6^	-	300	100	18	[49]
200	3.6 × 10^−6^	350	100	2.14	This work

## Data Availability

Not applicable.
